# Unraveling the Balance between Genes, Microbes, Lifestyle and the Environment to Improve Healthy Reproduction

**DOI:** 10.3390/genes12040605

**Published:** 2021-04-20

**Authors:** Valeria D’Argenio, Lara Dittfeld, Paolo Lazzeri, Rossella Tomaiuolo, Ennio Tasciotti

**Affiliations:** 1 Department of Human Sciences and Quality of Life Promotion, San Raffaele Open University, via di Val Cannuta 247, 00166 Roma, Italy; tasciottiennio@gmail.com; 2 CEINGE-Biotecnologie Avanzate, via G. Salvatore 486, 80145 Naples, Italy; 3 IRCCS San Raffaele Pisana, Via di Val Cannuta 247, 00166 Roma, Italy; 4 Aella Labs GmbH, Ra Curta 1, 6967 Lugano, Switzerland; lara.dittfeld@gmail.com (L.D.); plazzeri@me.com (P.L.); 5 Università Vita-Salute San Raffaele, Via Olgettina 58, 20132 Milano, Italy; tomaiuolo.rossella@hsr.it

**Keywords:** human reproduction, infertility, genetic factors, microbiome, lifestyle, environment

## Abstract

Humans’ health is the result of a complex and balanced interplay between genetic factors, environmental stimuli, lifestyle habits, and the microbiota composition. The knowledge about their single contributions, as well as the complex network linking each to the others, is pivotal to understand the mechanisms underlying the onset of many diseases and can provide key information for their prevention, diagnosis and therapy. This applies also to reproduction. Reproduction, involving almost 10% of our genetic code, is one of the most critical human’s functions and is a key element to assess the well-being of a population. The last decades revealed a progressive decline of reproductive outcomes worldwide. As a consequence, there is a growing interest in unveiling the role of the different factors involved in human reproduction and great efforts have been carried out to improve its outcomes. As for many other diseases, it is now clear that the interplay between the underlying genetics, our commensal microbiome, the lifestyle habits and the environment we live in can either exacerbate the outcome or mitigate the adverse effects. Here, we aim to analyze how each of these factors contribute to reproduction highlighting their individual contribution and providing supporting evidence of how to modify their impact and overall contribution to a healthy reproductive status.

## 1. Introduction

Our health is determined by the contribution of intrinsic and extrinsic factors that affect the way our cells, tissues, organs and body work as an integrated system. Similarly, many pathological conditions affecting the proper functioning of our body are also determined by a mixture of causes, generally ascribed to congenital and external elements. Dissecting what has the greatest effect and what can be changed or not has been an ongoing debate and is probably one of the key challenges scientists are facing today.

For many years, researchers have focused on the impact of our genetic code on health and disease. Since the completion of the human genome program in 2003 [1], we have spent a great deal of resources in understanding the role of single genes and the relative contribution of multiple genes in the determination of a particular phenotype. As of today, we have a much deeper understanding of the function of many individual genes and a better comprehension of their involvement in the definition of complex traits, but we are still far from having a complete picture of the causes and effects that link our genetic background to the multitude of possible outcomes that humans face in their lives. Gene expression and regulation, as well as the interaction between multiple genes still remain debated questions that will require more studies. The advent of faster sequencing technologies together with the computational power offered by artificial intelligence and machine learning have opened a new frontier in genetic studies and offered the promise to greatly advance the field in the years to come [2,3].

Concomitantly, a growing interest has been devoted to explaining how the environment around us affects our biology, through its interplay with the genetic determinants that govern the cellular mechanisms at the basis of human physiology. External environmental factors, like food, drugs, chemicals, temperature, and light, can influence gene expression and determine which genes are turned on and off, thereby affecting the way our organism develops and functions. This has spurred a flourishing interest in assessing how lifestyle and life choices can alter the genetic program written in our cells, and has resulted in a growing body of evidence that supports the overall claim that we are the product of both “nature and nurture” [4]. With the first, we refer to the overall predetermination of our fate as it is influenced by genetic inheritance and other biological factors. With the latter, we allude to the influence of external factors that result from what we are exposed to, what we experience and what we choose.

More recently a third element has appeared on the scene and has quickly gained momentum in the research laboratories around the world: our microbiome. It is now common knowledge that the collection of microbes that live on and inside us has the ability to deeply affect how we function by regulating several molecular mechanisms that ultimately influence the performance of cells and tissues. Microbes can alter our metabolism by secreting a plethora of factors that have the ability to crosstalk with the molecular machinery of our own cells. These factors can either contribute to the maintenance of our body or wreak havoc and short circuit our homeostasis [5]. As fundamental new studies continue to appear, it becomes more and more obvious that many explanations that were not possible by leveraging what we knew about genetic or environmental factors could be given leveraging the role of this third player, microscopic in size but mightily powerful in scope.

**Figure 1:**
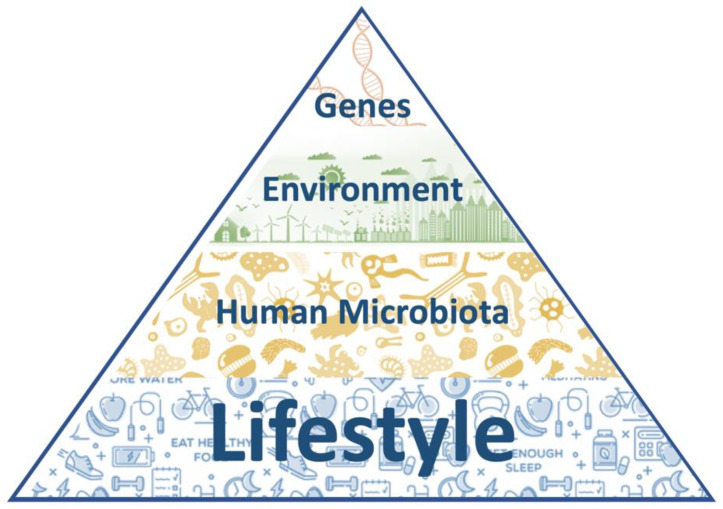
The nature and nurture pyramid. At the base of the pyramid there are the most modifiable factors (lifestyle habits and the microbiota composition), while the less modifiable ones rise towards the apex (environmental factors and the individual genetic background).

As the knowledge of the inner working of our organism grows, so does our understanding of the pathologies that can affect it. The relative impact of each of the above-mentioned factors on several diseases and complex ailments has been analyzed and today we have a more comprehensive roadmap to address the origin of several diseases and to reach a better prevention, fuller diagnosis and more effective treatment (Figure 1). 

In this review, we examine the impact of these factors on one of the most crucial functions of our body: reproduction. What is considered as one of the key elements to identify a living being (i.e., the ability to generate a progeny) has been alarmingly declining in western civilization. Several factors seem to have put at risk such a fundamental function and this topic has generated a great deal of attention in the scientific community.

## 2. Materials and Methods

Indexed articles written in English and published within 2010 and 2021 were searched in Pubmed. The following keywords were used for articles retrieval: “genetics and infertility”, “autoimmunity and infertility”, “female infertility and microbiota”, “male infertility and microbiota”, “pollution and human reproduction”, “endocrine disruptors and human reproduction”, “physical activity and fertility”, “sleep and fertility”, “ and “nutrition and fertility”. Moreover, the oldest references within the resulting articles were manually searched.

## 3. Intrinsic Factors

### 3.1. Medical Conditions

#### 3.1.1. Genetic Diseases

The genetic conditions related to infertility, including the common and rare ones, account for almost 50% of all infertility causes [6,7]. In the presence of high suspicion of a genetically based infertility (such as malformations, recurrent abortions, and family history), according to the signs and symptoms observed by the specialist during the medical examination, a genetic test can provide a more accurate diagnosis of infertility and inform the couple about the risk of transmission of genetic defects to the offspring [8]. 

In men, alterations in the standard semen analysis are the first indication for genetic tests, particularly in cases of severe oligospermia (<5 million/mL) [9]. Although genetic factors have been identified in all the etiological categories of male fertility (pre-testicular, testicular and post-testicular), the main genetic tests routinely used for the diagnosis of male infertility are limited to the karyotype, the study of chromosome Y microdeletions and the analysis of the *CFTR* gene [6]. Genetic disorders related to male infertility include whole chromosomal aberrations (structural or numerical), partial chromosomal aberrations (i.e., microdeletions of the Y chromosome) and monogenic diseases [10]. In particular, abnormalities in sex chromosomes have a greater impact on spermatogenesis, while mutations affecting autosomes are more related, for example, to hypogonadism, teratozoospermia or asthenozoospermia and to familial forms of obstructive azoospermia. Klyneferter syndrome (47, XXY) and Double Y syndrome (47, XYY) are the most frequent chromosome aneuploidies related to male infertility [11,12]. Individuals carrying these chromosomal alterations not only have a reduced fertility, but also shows an increased risk of abortion and having a child with karyotype alterations [6]. Among the partial chromosomal alterations, microdeletions in the long arm of Y chromosome, involving the so-called azoospermia factor (AZF) region, are the most common genetic causes of male infertility [13]. Indeed, the AZF region includes genes involved in the spermatogenesis, so that their deletion is related to an impaired reproductive capacity. In addition to chromosomal aberrations, more than 200 genetic conditions related to male infertility are reported in the Online Mendelian Inheritance in Man database (OMIM), ranging from the most common clinical presentations of infertility to the rarest complex syndromes [14]. The search for pathogenic mutations in one or more genes should be evaluated based on patients clinical phenotypes. For instance, congenital hypogonadotropic hypogonadism (CHH) is a rare endocrine disease featured by a deficient gonadotropin-releasing hormone (GnRH) activity due to both defective synthesis or peripheral resistance [15]. CHH clinical phenotypes range from complete and more severe forms with the absence of puberty, to late-onset hypogonadism. To date, more than 30 genes have been related to this condition and their testing should be considered in male with CHH, after the exclusion of secondary forms [16]. Similarly, the congenital absence of vas deferens (CAVD) may be both an atypical presentation of cystic fibrosis or an isolated reproductive disease. Thus, the *CFTR* gene mutations screening is recommended in male patients with CAVD and, since novel candidate genes are emerging for the isolated forms, it may be useful to enlarge the molecular screening to include them [17]. Altered sperm features, as assessed by semen analysis, may be due to rare diseases inherited as recessive traits. Within this category: macrozoospermia is a condition featured by large-headed and multiflagellated spermatozoa due to alterations of spermatozoa meiotic division; globozoospermia is a rare disease characterized by round-headed spermatozoa without acrosome; acephalic spermatozoa is a rare disease featured by the presence of headless spermatozoa; and multiple morphological abnormalities of the sperm flagella is another rare condition featured by morphological alterations affecting sperm flagella [16]. One or more causative genes have been identified for all these rare inherited diseases. Their testing should be considered based on semen parameters [18,19,20,21]. Moreover, the Kartagener syndrome or primary ciliary dyskinesia is a rare genetic disease featured by abnormal internal organs position, high frequency of respiratory infections and asthenozoospermia, as a consequence of motility defects of both cilia and flagella [22]. In this disease, sperm analysis usually doesn’t show morphological alterations, but spermatozoa have several structural abnormalities due to dyneins loss and microtubular rearrangements. About 30 genes have been related to Kartagener syndrome, *DNAI1* and *DNAH5* accounting for up to 30% of cases [23,24]. Mutations in the cation channel of sperm (*CATSPER*) genes cause asthenozoospermia due to the incapacity of sperm to undergo hyperactivated motility during sperm capacitation [25]. Additionally, androgen receptor (*AR*) mutations have been related to male infertility issues [26]. To date, more than 1000 *AR* mutations have been identified and associated with different phenotypes of androgen insensitivity syndrome, ranging from severe to mild forms [26]. Finally, novel candidate genes are emerging due to the diffusion of next generation sequencing-based analyses and whole exome sequencing screening. Once the effects of these genes (and consequently of their mutations) in reproduction will be functionally assessed, their molecular testing may improve infertile men clinical management [16].

In females, fewer specific tests are routinely recommended to identify chromosomal and genetic alterations that could interfere with healthy reproduction, i.e., karyotype analysis and genetic test for *FMR1*/*FMR2* (Fragile X Mental Retardation 1 and 2) are advisable in case of fertility impairment. The *FMR1* premutation (the number of CGG repeats falls between 55 and 200) or *FMR2* microdeletions are related in females with menstrual dysfunction, diminished ovarian reserve and premature ovarian failure [27,28]. Several chromosome aberrations have been associated with female infertility, which primarily involves oogenesis. Turner syndrome (45, X0) and X chromosome cytogenetic alterations, including both reciprocal (exchange of two-terminal segments from different chromosomes) or Robertsonian (centric fusion of two acrocentric chromosomes) translocations, can cause blockage of meiosis resulting in primary ovarian insufficiency. In particular, reciprocal translocations are related to a significantly increased risk of infertility (i.e., hypogonadotropic hypogonadism with primary or secondary amenorrhea or oligomenorrhea), as balanced rearrangements can become a cause of multiple miscarriages [29]. Thousands of genes are involved in human reproduction, about 200 of which have been related to infertility since they are able to affect specific steps required to this process [30]. For instance, genetic causes of gonadal disgenesis have been identified. In this context, the Swyer syndrome is a defect of sex determination occurring in XY individuals showing a female phenotype with gonadal dysgenesis, absence of pubertal development, primary amenorrhea and infertility [31]. Several molecular alterations have been related to this syndrome: about 15% of the patients carry pathogenic mutations in the *SRY* gene but Y chromosome structural alterations, or mutations in other genes, such as *NR5A1*, *NR0B1*, *WNT4*, *AR*, *MAP3K1*, *GATA4*, *DMRT1*, *DMRT2*, *ZNRF3*, and *DHH*, have been also reported [30]. Another gonadal alteration is the ovarian dysgenesis occurring in XX individuals showing an impaired ovarian development. About 10 different genes have been implicated in this process and their defective functions may impair gonadal development leading to the onset of a wide spectrum of clinical phenotypes, including isolated and syndromic conditions, associated with complete gonadal dysgenesis and less severe forms of primary ovarian insufficiency [32,33]. Further, defects of early oogenesis have been related to female infertility. Indeed, an increased cell death rate causes the depletion of the follicle pool, incomplete follicles development, altered sexual differentiation and gonadal dysgenesis. Mutations in more than 30 genes implicated in meiosis, germ cells mitosis and DNA damage repair have been described as related to oogenesis alterations and their testing may be evaluated based on patients clinical signs taking into account that these mutations have been identified in patients with idiopathic infertility and a positive history for recurrent abortions [30,34]. Moreover, it is well established that chromosomal segregation errors during meiosis occur more frequently with the increase of women age [35]. These age-related aneuploidies, and consequent infertility, have been associated with the progressive loss of cohesins proteins, such as SGO2 [36]. Mutations in genes involved in mitotic checkpoints, like *BUB1B* and *CEP57*, can lead to multiple chromosomal alterations resulting in defective oocytes maturation, embryonic death and miscarriages [37,38]. Several studies have highlighted the role of genes involved in DNA repair in follicles maturation and quality, reproductive aging and the age at menopause [30]. Indeed, these genes play a role both in meiosis and mitosis and can cause variable phenotypic expression, including syndromic conditions featured by growth retardation, developmental defects, endocrine disorders, gonadal alterations and increased susceptibility to cancers development [30]. In addition, mutations in *MCM8*, *MCM9*, *XRCC4*, and *MSH5* are able to induce a non-syndromc primary ovarian insufficiency [39]. Altered folliculogenesis is another pathogenetic mechanism underlying female infertility and also in this case several genetic alterations have been identified so far. Indeed, a woman’s reproductive life depends on the number of primordial follicles, their quality and germ cell depletion [39]. *GDF9* and *BMP15* gene variants, have been identified in about 10% of women with hypergonadotropic ovarian failure, primary ovarian insufficiency, amenorrhea, and polycystic ovary syndrome [40]. Similarly, mutations in multiple oocyte-specific transcription factors (such as *FIGLA*, *NOBOX*, *LHX8*, *SOHLH1*, and *SOHLH2*), being involved in follicular development and future embryonic activation, have been found to be associated with ovarian dysgenesis and primary ovarian insufficiency [32]. Interestingly, primary ovarian insufficiency has been described in different syndromic conditions, such as Perrault syndrome, epicanthus inversus syndrome, blepharophimosis with ptosis, leukoencephalopathy with vanishing white matter, galactosemia and carbohydrate-deficient glycoprotein syndromes [30]. Moreover, also mutations in the mitochondrial *POLG* gene have been identified in women with primary ovarian insufficiency [41]. Folliculogenesis, oocytes maturation, ovulation and implantation are regulated by the action of the follicle-stimulating hormone (FSH) and luteinizing hormone (LH); thus, mutations affecting their corresponding genes are able to lead to fertility impairment. Indeed, women carrying mutations in *FSHB* and *FSHR* genes result respectively in hypogonadotropic and hypergonadotropic hypogonadism [42,43]. Hypogonadotropic hypogonadism is a rare disease due to GnRH deficiency resulting in incomplete or absent puberty and infertility, and associated with more than 25 causative genes [44,45]. Furthermore, female-specific factors affecting genes involved in sperm capacitation and the sperm’s ability to penetrate the zona pellucida cause fertilization failure and infertility [46]. Finally, as for male, next generation sequencing approaches are allowing the identification of an increasing number of genetic variants associated with female infertility, thus suggesting their possible use as genetic biomarkers for infertility. 

Although our knowledge of infertility’s molecular bases is continually growing, genetic tests for male and female infertility suffer from an ineffective approach in clinical practice. An in-depth analysis using a targeted genetic test, chosen after an accurate evaluation of the medical and familial history, could identify a specific genetic disease thus allowing a personalized diagnostic and therapeutic management (i.e., fertilization with donor, preimplantation genetic diagnosis, etc.) [8]. To date, the development of sequencing technologies has encouraged the use of gene panels, which have proven helpful companion diagnostics for different pathologies [47,48,49]. The European Society of Human Genetics (ESHG) and the European Society of Human Reproduction and Embryology (ESHRE) have recently issued a recommendation for the introduction of targeted multigene panels, as expanded carrier screening [50,51]. Genetic tests based on parallel sequencing of several genes facilitate the process of gene investigation in infertility, reducing diagnostic costs and time [8], decreasing the current 20% rate of idiopathic infertility, and characterizing the different subtypes of male and female infertility [7,52]. 

The general state of health in reproduction is gaining increasing attention and clinical relevance. Therefore, infertile couples must be evaluated considering the aspects of public and psychological health, as well as the reproductive element, since the relative conditions of comorbidity can influence their reproduction. This will allow changing couples’ management, moving from a population-based view to an individual-based one. For example, numerous studies show that the difference in the response to therapy found among patients, may be due to specific DNA variations; thanks to genetic characterization, the clinicians are now able to choose the most appropriate approach for the prevention or treatment of the condition in individual patients and infertile couples [17,53,54].

Outside of particular hereditary diseases, the idea of a single “responsible gene” for traits or diseases is rarely viable. In most cases, there are hundreds, thousands of genes that contribute to a complex trait, such as reproduction. To identify fertility-related genetic variants and genomic loci, more than 70 genome-wide association studies (GWAS) have been carried out analyzing multiple samples and more than 30 traits associated to reproduction have been found [55]. In this context, Barban et al. [56], analyzing about 700,000 individuals, identified 12 loci associated with reproductive behavior highlighting also candidate genes and potentially causal variants [56]. Recently, Loizidou et al. [57] assessed in a Ukainian cohort an association between 12 genetic loci and recurrent pregnancy loss [57]. In addition to the possibility to discover novel candidate genes and mutations, these GWASs on common genetic risk factors provide novel clues to interpret the underlying relationships and causality involved in the regulation of reproduction and fertility. These data will allow to achieve new insights into disease risk, disease classification and co-morbidity for many diseases associated with reproduction alterations and infertility. Moreover, while the study of genetics has brought under the spotlight the importance of gene mutations in the probability of procreating, other factors have also been considered as pivotal and have gained the attention of several studies in the scientific community.

#### 3.1.2. Autoimmunity

Autoimmune diseases (ADs) are characterized by multi-system involvement, and a mounting body of evidence points to their impact on both fertility and pregnancy. ADs are easily overlooked as they can be clinically silent or present with non-specific symptoms and continue to remain undiagnosed until a severe complication is encountered. ADs affect different stages of female fertility, such as ovarian reserve, fertilization, and implantation. About 10–30% of women with premature ovarian failure have an AD, the most common being systemic lupus erythematosus (SLE), rheumatoid arthritis, and autoimmune thyroid diseases [58]. In these disorders, autoantibodies are produced against steroid-producing cells resulting in oophoritis and immune cells infiltration of pre-ovulatory follicles and corpus luteum [58,59]. In SLE, a prolonged inflammation causes dysfunction of the hypothalamus-pituitary-ovarian axis resulting in menstrual irregularities; moreover, SLE medical therapies, such as high dose of steroids and immunosuppressive drugs, also impair fertility [58,59].

Auto-immune thyroid diseases are characterized by high titers of anti-thyroglobulin and anti-thyroid peroxidase antibodies. Monteleone et al. [60] demonstrated the presence of thyroid autoantibodies in the follicular fluid, and their levels strongly correlated with serum concentrations. They proposed that these antibodies bind to antigens expressed in the zona pellucida, damaging the maturing oocytes, and reducing both the fertilization and implantation rates [60]. A meta-analysis evaluating the impact of thyroid autoimmunity on in vitro fertilization (IVF)/intracytoplasmic sperm injection (ICSI), found that the fertility rate was not affected suggesting that ICSI could overcome the negative impact of thyroid autoantibodies [61].

Auto-immune mechanisms have also been described in endometriosis, a well-known condition associated with infertility. Indeed, IgG antibodies directed to laminin-1, and thus affecting the implantation process, have been found in patients with endometriosis [58,62]. Similarly, antiphospholipid antibodies (APL) also affect implantation, placentation, and early embryonic development, thus impairing fertility [58,62]. Finally, ADs like type 1 diabetes mellitus (DM) have been associated with polycystic ovary syndrome (PCOS) that reduces fertility by causing multiple endocrine dysfunctions [58].

ADs have been reported to affect also pregnancy outcomes, recurrent pregnancy loss (RPL) being one of the dreaded complications of ADs. Mumusoglu et al. [63] observed a significantly higher frequency of RPL in the autoantibody-positive women than controls, and the RPL ratio positively correlated with autoantibodies concentration [63]. Among the different autoantibodies, antiphospholipid antibodies (APL) showed a higher association with RPL. The degree of association varies with the type of APL, among which Lupus anticoagulant and IgG anticardiolipin antibodies were found to be the most significant [63]. It has been proposed that this phenomenon may be due to the ability of APL to disrupt adhesion molecules between the trophoblast cells, to damage the trophoblast through the action of cytokines, and to increase the risk of placental thrombosis [58,64]. An increased risk of miscarriage and RPL in women with positive thyroid autoantibodies has been demonstrated in both natural and IVF pregnancies [61,62]. Indeed, the generalized immune imbalance and the diminished thyroid reserve caused by thyroid autoantibodies decrease the ability of the thyroid gland to adapt to the physiological changes due to pregnancy. These effects are amplified in IVF pregnancies due to the harmful impact of ovarian stimulation on thyroid function. Late pregnancy complications have been also associated to ADs, including eclampsia, oligohydramnios, intrauterine growth restriction, stillbirth, and preterm deliveries [59,62,63,64]. Moreover, anti-SSA and anti-SSB antibodies are associated with neonatal lupus and isolated congenital heart block in babies born to mothers with ADs [59,64].

Though ADs are more common in women, also men’s fertility may be affected by these disorders. In particular, anti-sperm antibodies (ASA) are auto-antibodies targeting the seminiferous tubules and are responsible for autoimmune orchitis. ASA are able to affect several sperm features, including sperm motility, penetration of cervical mucus and migration through tubes. Moreover, they affect sperm capacitation and acrosome reaction, thus interfering with sperm-oocyte interaction and ultimately preventing the implantation of the embryo and its further development [65]. In addition to ASA, secondary orchitis caused by testicular vasculitis observed in other ADs, like SLE, rheumatoid arthritis and polyarteritis nodosa, may have similar effects on male infertility [65]. 

Knowledge about the impact of ADs on fertility is essential since their complications are preventable with timely diagnosis and appropriate therapy. As ADs are notable for silent and/or non-specific clinical signs, a high index of suspicion helps to diagnose them at an early stage. Early diagnosis provides the benefit of initiating appropriate treatment and close monitoring to prevent potential complications.

### 3.2. Human Microbiota

A growing body of evidence suggests the critical role of microbes in the acquisition and maintenance of human health [5,66,67]. To date, bacteria have been identified in almost all body niches and several functions required for human homeostasis have been assigned to them. Accordingly, microbial alterations have been associated to an increasing number of diseases [68,69,70]. These findings have increased the interest into the study of the human microbiota since, in addition to providing novel clues related to the pathogenetic mechanisms underlying specific diseases, it may represent an actionable target for specific therapies.

In this scenario, a role of the microbiota in human reproduction has emerged [71,72]. In particular, the semen microbiota composition has been related to altered sperm cells motility, hyperviscosity, oligoasthenoteratozoospermia, and/or sperm DNA fragmentation [73,74,75]. Indeed, microbes can exert dangerous effects on sperm cells using different mechanisms, i.e., cytokines or reactive oxygen species induction, cellular adhesion or soluble factors production [76,77,78]. It is important to underline that semen microbiota has shown a high inter-individual variability among healthy donors [79]. Indeed, several factors, including the geographical location, diet, age, hygiene practices, circumcision, age at sexual debut and sexual activities, are able to influence the semen microbiota composition [72]. In this scenario, *Lactobacillus*-predominant semen has been associated to improved semen parameters [80,81], while *Ureaplasma urealyticum*, *Enterococcus faecalis*, *Mycoplasma hominis* and *Prevotella* negatively impact semen quality [82]. Even if these studies present some limitations and need deeper investigations, the association between semen microbiota and infertility appears to have some merit. Moreover, through the transfer of microorganisms, the semen microbiota seems to be able to influence also couples’ and offspring’s health [73,83,84,85].

Similarly, the female reproductive system microbiota has been related to fertility, pregnancy establishment and maintenance [86,87]. Several studies have assessed that infertile women harbor a different microbiota in the upper and/or lower reproductive system, compared to fertile women [88,89,90,91,92]. The female reproductive system’s microbiota can influence women’s health, and through the transmission of microbes between partners, also impact the partners’ health and fertility. Indeed, it emerged that the evaluation of both partners’ microbiota, the so called “seminovaginal microbiota”, is crucial to have a proper assessment of the couple’s fertility status [71]. Based on all the above, it became crucial to understand what are the factors able to modify the male and female reproductive system’s microbiomes in order to improve couples fertility [93,94].

The *Lactobacillus* is the most abundant genus in the female reproductive system microbiota, and its reduction is usually associated to pathological conditions [71]. As for the case of semen microbiota, several factors are known to be able to modify the female reproductive system microbiota. The vaginal microbiota, in particular, is affected by physiological fluctuations due to sex hormones activity and reproductive age. Moreover, hygiene habits, sexual exposure, use and type contraceptives, and change of sexual partners have been associated to microbial modifications over time [71]. It is well known that both gut and vaginal microbiotas change during pregnancy, and these modifications are believed to induce immune tolerance in the mother [95,96,97]. A very recent work by Di Simone et al. [98] reviews the impact of maternal microbiota on pregnancy outcomes highlighting that: (i) maternal gut microbiota may impact the development of autoimmune and lifelong diseases in the unborn; (ii) vaginal microbiota modifications, i.e., the reduction of *Lactobacilli* and an increased bacterial diversity, are associated to pregnancy complications, including preterm birth; and (iii) the endometrium hosts its own microbiota whose alterations may impact both fertility and pregnancy outcomes [98]. In addition, alterations of both vaginal [99] and endometrial microbiota [100,101,102,103] have been found to be negatively correlated to IVF pregnancy rate. A prospective study carried out on 300 women in reproductive age and candidate to an IVF procedure validated the predictive value of vaginal microbiome assessment on IVF outcome, suggesting the introduction of this kind of evaluation in diagnostic algorithms [104].

The possibility to use therapeutic approaches able to target the microbiota, restoring a more physiological situation, and thus positively impact couples fertility and pregnancy outcomes is a topic of increasing interest in the management of these patients. Plummer et al. [105] showed that treating with antimicrobial drugs the male partners of patients with bacterial vaginosis reduced the disease recurrence, stressing the need for coordinated therapy for couples [105]. The use of antibiotics in patients with chronic endometritis has also proven its positive effects on IVF outcomes and on spontaneous pregnancy rates and live birth rates [106,107]. 

The use of probiotics is another attractive intervention in this field and several oral and vaginal probiotics, usually containing *Lactobacillus* spp., are available on the market [71]. Bacterial vaginosis is a common form of vaginal dysbiosis that is known to negatively impact both fertility and pregnancy outcomes [108]. Probiotics, such as *L. reuteri RC-14*, *L. fermentum*, *L. gasseri*, *L. rhamnosus*, *L. acidophilus*, *L. crispatus*, *L. casei*, and *L. salivarius*, are commonly used to treat this condition, avoiding antibiotic abuse and exerting positive effects on fertility [109,110,111,112,113]. A debating issue is the administration route of these probiotics. Oral administration is the most common route but to be beneficial probiotics are required to be resistant to the low gastric pH and intestinal hydrolytic activities and to be transferred to the body site where they are expected to act. Given these issues, vaginal administration may be preferred in some conditions [108]. 

López-Moreno and Aguilera recently reviewed the modulation of fertility-related dysbiosis through dietary probiotics supplementation in women [108]. They found more than 700 studies, 222 fulfilling their selection criteria and 10 being clinical trials, showing potential benefits of these treatments. However, the high heterogeneity regarding the selection of probiotic strain, doses, administration pattern and key endpoints modulation capacities underlines the need for more standardized protocols [108]. Probiotics supplementation has been reported to exert beneficial effects also on the quality of semen [72]. A six-week supplementation with *Lactobacillus* and *Bifidobacterium* in asthenozoospermic males has shown its efficacy by increasing sperm motility and reducing the rate of sperm DNA fragmentation [114]. Moreover, a six-months treatment with a daily administration of *Lactobacillus paracasei*, arabinogalactan, fructo-oligosaccharides, and l-glutamine had a positive effect on sperm count and motility, while atypical forms where reduced, when compared to the placebo-receiving group [115]. 

Finally, the administration of prebiotics may also have beneficial effects in the management of the gut microbiota of infertile couples. Komiya et al. [116] compared infertile and fertile-control women and highlighted an increased abundance of the *Verrucomicrobia* phylum in the patients with infertility [116]. Moreover, in a subgroup of patients, they were also able to evaluate the effects of the oral administration of partially hydrolyzed guar gum on gut dysbiosis and on pregnancy outcome of assisted reproductive technology. Interestingly they found a success rate of 58.3% in the patients who received this combined therapy and observed in the gut microbiome a reduction of *Paraprevotella* and *Blautia* and an increase of the *Bifidobacterium* genus, suggesting that this oral prebiotic supplementation was able to restore the gut dysbiosis and improve the success rate of pregnancy in infertile women [116].

Even if most of these studies still present several limitations related to the small number of patients analyzed, the chosen methodologies and the lack of harmonized protocols to evaluate the efficacy over time of the proposed interventions, they have the merit to highlight an overall improvement of fertility. In the future, larger and better designed interventional studies will allow to definitively assess the advantages of prebiotics and/or probiotics administration in infertile couples with possible clues for personalized treatments.

## 4. Extrinsic Factors

### 4.1. Pollution

#### 4.1.1. Environmental Pollution

The environment in which we live acts in a profound way on our physiology, so we have to worry about both the external environment and how it affects the internal one. The impact of environmental pollution on fertility (both male and female) is a topic that has sparked a growing interest in recent years and numerous studies have been carried out on it. While it is often easy to find contradictory research on any given topic, there is substantial convergence on the fact that pollution can cause significant damage to fertility. In 2017, an international team of scientists published a meta-analysis of more than 7500 studies carried out on 43,000 men living in Western countries, showing that sperm concentration dropped by more than 50% in just under 40 years (the period covered goes from 1973 to 2011) [117].

**Figure 2:**
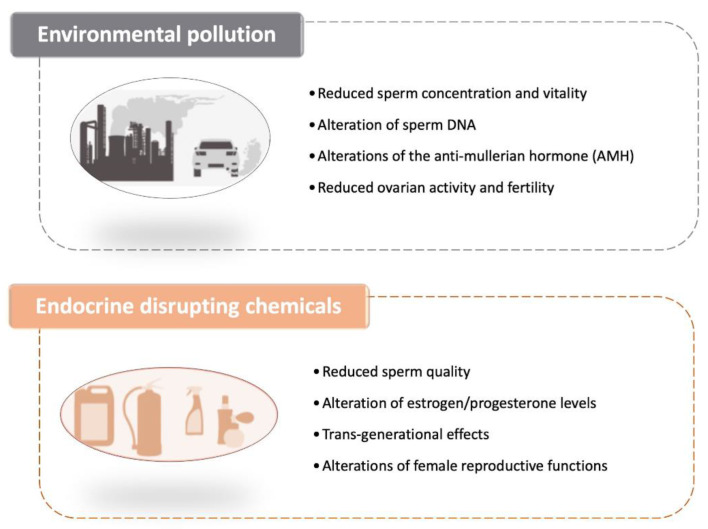
Effects of environmental pollution and endocrine disrupting chemicals on fertility [117,118,119,121,122,127,128,129,130,133,142,143].

Because of the significant public health implications of these results, research on the causes of this continuing decline has increased over the past few years. The negative impact of environmental pollution such as that caused by the presence of heavy metals in the air (such as those grouped under the wide umbrella definition of PM2.5–PM10) on the vitality, quality and motility of spermatozoa has now been established as one of the main culprits (Figure 2) [118]. 

A recent study has gone beyond the number, vitality and mobility of sperms and has revealed that there are some pollutants that can even modify their DNA structure. It follows that it is not only the subjects exposed to pollutants who are most vulnerable to certain pathologies, but also the future generations, as they are less likely to be procreated [119]. It is also interesting to note that the seminal fluid displays an accumulation of these substances (heavy metals and other pollutants), whose presence cannot be measured at a significant dose in the blood of the controlled subjects. It follows that sperm analysis could be exploited as a biomarker of environmental exposure and perhaps serve as a more effective indicator than other environmental chemical or laboratory analyses [120].

For what pertains female infertility, animal experiments have previously shown that pollution could affect the level of the anti-mullerian hormone (AMH), the hormone released by the cells of the ovaries which indicates their level of fertility [121]. A recent study has indicated that air pollution could be linked to a decrease in the activity of the ovaries and a reduction in fertility in women. The study examined AMH levels in about 1300 women over a 10-year period, and showed that the female reproductive system was affected by environmental exposure to pollutants, even if it is not yet possible to specifically assess what was the molecular and cellular mechanisms at the basis of this linkage. Levels of AMH in the blood tend to decline over time for women over 25 years of age. Having a high level of AMH is a reproductive advantage, as women with high levels of AMH have a longer fertile period and more eggs, which may lead to more embryos. The team found that AMH levels were lower in women who lived in areas with higher levels of pollution as assessed by measuring the daily values of fine particles (PM 2.5 and PM 10). When the team divided air pollution into four brackets, they found that three times as many women living in the lower bracket had lower levels of AMH than those in the higher brackets, referring to a level below 1 ng/mL, a value that correlates with a serious ovarian deficiency [122] (Figure 2).

While the link between AMH levels and the possibility of getting pregnant is still under debate, the findings suggest that environmental factors play a vital role in the female reproductive system. There remains a question to be answered: if the effects of air pollution on female and male fertility are permanent or only temporary, and if full reproductive functionality can be recovered once we leave the polluted areas. 

#### 4.1.2. Endocrine Disrupting Chemicals

Dr Maria Neira, WHO’s Director for Public Health and Environment, has recently declared that “we urgently need more research to obtain a fuller picture of the health and environment impacts of endocrine disruptors” [123]. Indeed, environmental reproductive health focuses on the exposures to environmental contaminants, a daunting proposition considering that over 87,000 different chemicals are in use today. Even if very few of these have been tested for their endocrine effects [124], it has been estimated that 800–1000 of these could be endocrine disruptors [125].

Endocrine disrupting chemicals (EDCs) are natural or synthetic compounds able to modify the hormonal and homeostatic systems as a consequence of an environmental or inappropriate developmental exposure [126]. To date, EDCs have been associated with reduced fertility both in men and women, higher incidence of endometriosis and menstrual pain, PCOS, and thyroid alterations (Figure 2). Moreover, they can also affect the quality of sperm thus reducing the chances of conceiving [127]. For example, the presence of EDCs, such as phthalates, bisphenol-A and polychlorinated biphenyls (PCBs), in the blood and urine of men is associated with a lower quality of seminal fluid in terms of number, motility and morphology of spermatozoa [128,129].

Even if further studies are needed in order to better understand the complex mechanism by which these substances act and interfere with the normal biology and functioning of the human body, it is now clear that they act via nuclear receptors, non-nuclear steroid hormone receptors (e.g., membrane estrogen receptors—ERs), nonsteroid receptors (e.g., neurotransmitter receptors such as the serotonin, dopamine, or norepinephrine receptors), orphan receptors (e.g., aryl hydrocarbon receptor), enzymatic pathways involved in steroid biosynthesis and/or metabolism, and numerous other mechanisms that converge upon endocrine and reproductive systems [126]. Many of the products that are used on a daily basis, whether for home cleaning or personal care, and even food contain EDCs. When absorbed by the body, EDCs can reduce or increase normal hormone levels, mimic hormones, or alter natural hormone production. In particular, their presence in biological fluids (blood, seminal fluid and follicular fluid) raises great concerns about their possible effect on fertility. For example, they can negatively affect the balance between estrogen and progesterone, which is necessary for ovulation, fertility and pregnancy [130]. Within EDCs, there is a wide range of highly heterogeneous substances, both natural and artificial, that can alter the endocrine system, including phytoestrogens, phthalates, dioxins, pesticides and plasticizers. Everyday products, like cosmetics, perfumes, personal care products, cleaning products, detergents, non-stick cookware, toys, plastic bottles, pesticides, and even canned foods can contain this group of molecules [131]. 

Usually, industrialized areas are contaminated by several industrial chemicals that may be present in the soil and/or in the groundwater. These complex mixtures enter the food chain and accumulate in animals higher up the food chain. Some endocrine disruptors may also persist in the environment as they are not biodegradable and their effects on human health can be due to their bioaccumulation. DTT (dithiothreitol) and PCBs, for example, whose use was banned decades ago due to their harmful effects, are still present in the blood of people and animals living in the areas where they were once used. Other substances which are less persistent in the environment, can still act as endocrine disruptors following constant, repeated and prolonged exposure [131]. A high individual exposure to one chemical is often associated with a high exposure to other chemicals and the possibility of combination effects by multiple simultaneous exposures is very likely [132]. 

Since each individual has different physiology and reactions to these chemicals and exposure time and intensity vary, it is difficult to understand when a disorder is related to EDCs exposure. Moreover, there is usually a delay, a latency time, between exposure and manifestation of clinical symptoms. Despite this difficulties, it seems that the categories more at risk for EDCs exposure are pregnant women. In particular, several studies have correlated the exposure to EDCs already during intrauterine life to a higher incidence of abnormalities of the male reproductive system in the exposed population and a greater predisposition to develop testicular neoplasms [133]. Indeed, during prenatal, postnatal, as well as adult life, physiological oestrogen signalling and ERs’ expression in the male reproductive tract plays an important role [134]. The testis development, and indirectly the sperm production, is paired by physiological oestrogens’ level [135]. In addition, oestrogens affect sperm capacitation, thus being crucial for successful fertilization [136]. Interestingly, the alteration of sperm parameters, notably sperm counts, is paired with the oestrogen system disruption, in men that have been exposed to EDCs [137]. This effect was observed even in men whose fathers had been exposed to synthetic endocrine disruptors in utero [138]. Notably, an oestrogen-like response has been observed also after exposure to natural EDCs, i.e., oestrogen-like compounds produced by plants or fungi, named phytoestrogens; these EDCs become part of the food chain because fungi contaminate cereal crops [139]. Timing of exposure is determinant, but it has to be underlyined that EDCs have a very long half-life in the body as they are lipophilic and are able to bioaccumulate in the adipose tissue [140]. This represents a chemical body burden to which individuals respond differently depending on medical background, environmental and lifestyle habits [141].

In women, the exposure to EDCs during intrauterine life seems to affect their reproductive capacity. EDCs, by acting on folliculogenesis which begins as early as the 60th day of embryonic life, are responsible for the decrease of female fertility. Therefore, the effects of EDCs on human health are trans-generational, since they act both on the exposed population and on future generations [142]. Unlike spermatozoa, which are produced approximately every 70 days, the pool of female gametes is already determined at birth and can be negatively influenced in terms of both quantity and quality of oocytes by exposure to endocrine disruptors during pregnancy. In addition, it has been reported that EDCs exposure in women may interfere with all developmental stages of reproductive functions including puberty, menstruation and ovulation, fecundity, fertility, and menopause [143].

### 4.2. Lifestyle

**Figure 3:**
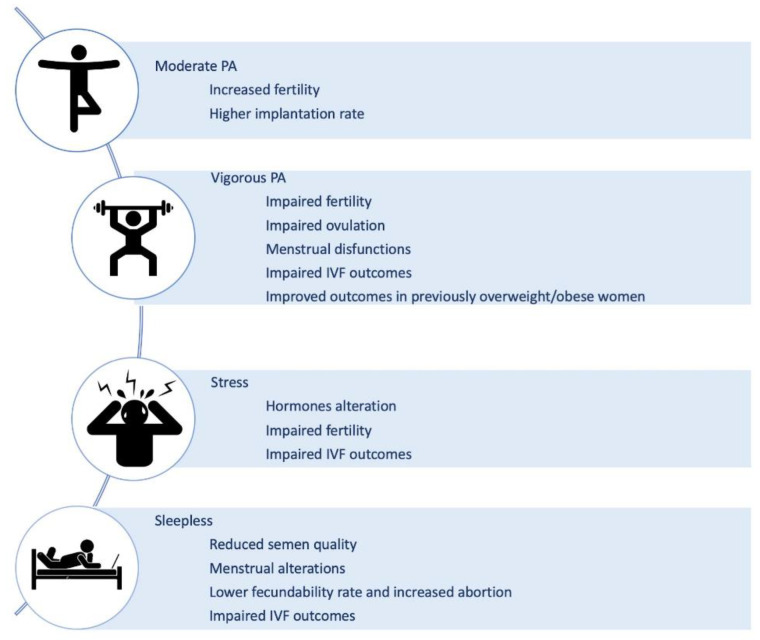
Lifestyle-related factors able to influence human reproduction. Physical activity (PA), stress and sleep have been related to different effects on both fertility and in vitro fertilization (IVF) procedure outcomes [145,146,147,148,151,156,157,158,159,160,161,164,165,166,167,170,173].

As mentioned in the previous sections, it is now well established that human fertility is influenced by a variety of factors acting through finely tuned mechanisms to allow reproduction (Figure 3). In particular, metabolic and lifestyle factors are gaining prominence in this context and will be reviewed in the next sections.

#### 4.2.1. Physical Activity

It is broadly established that the practice of physical exercise can positively affect weight and body composition, reduce the risk of numerous chronic diseases, preserve health and physical functions, and positively affect mood and mental balance [144]. However, the relation between fertility and physical activity (PA) is less clear. Although conflicting conclusions emerged from different studies, some common trends have emerged implicating an inverse association between vigorous PA and fertility, and a weak positive association between moderate PA and fertility [145]. This suggests that the effect of PA on fertility may be positive up to a certain level of activity, in terms of frequency and intensity, and then have a deleterious effect when that threshold is overcome (Figure 3). The difficulties in having univocal data are probably due to different types of infertility taken into consideration (anovulatory or idiopathic), lack of homogeneity in the definition of PA intensity (extremely heavy, vigorous, moderate), and not simultaneous evaluation of diet and energetic dietary balance.

The existing observational studies in the general population that aimed at measuring the effect of intense exercise on ovulation clearly showed that intense exercise has the capacity to disrupt ovulation. However, it has to be underlined that is the development of an energy deficiency, resulting from an energy intake that inadequately compensates for exercise energy expenditure, that can lead to menstrual dysfunction (Figure 3) [146]. 

In women who exercised 4 or more hours per week, negative effects have been related also to IVF outcomes. Indeed, women that have a regular routine of more than 4 h/weekly were 40% less likely to have a live birth (Figure 3) [147]. At the same time, women who were physically moderately active during treatment (pre and post-transfer) were more likely to have increased implantation rates and live birth results compared to no or low activity [148].

Despite these negative consequences, PA has shown to exert beneficial effects in some groups of patiens, like women with PCOS and obese women [149]. This occurs because the excess fat favors an increase in estrogen levels, following an increase in peripheral conversion—in particular by the fat tissue—from androstenedione to estrone, thus favoring a condition of anovularity. In overweight and obese women (independently from the presence of PCOS), exercise promotes a regular ovulation throught its ability to lower insulin and free androgen levels. In this context, it has been shown that obese PCOS patients with anovulatory infertility subjected to physical exercise for 24 weeks show a reduction in all obesity-related parameters, including insulin resistance, and resumed ovulation [150]. In a cohort study that involved 2062 women, moderate PA (between 1 and 5 h a week) was associated with increased fecundability, but there was no dose-response relation. Among overweight/obese women (BMI ≥25 kg/m^2^), fecundability was 27% higher for vigorous PA of ≥5 versus <1 h/week (Figure 3) [151]. In another cohort study, analyzing Australian women over a 15-years period, a lower risk of fertility issues was observed in the highly active and normal weight women, while a higher risk was observed in the obese women [152].

Similar observations are also being collected for male infertility. Indeed, male reproductive health can be improved by a proper PA, while both excessive intensity and duration of exercises may have dangerous consequences. Notably, the diffusion of the use of drugs to improve sport endurance, also by amatheur athletes, could impair the male hypothalamic-pituitary-gonadal axis, causing hypogonadism and infertility [153].

#### 4.2.2. Stress

For decades it has been known that stress can induce long-term changes in multiple neurochemical systems [154], causing the so-called “allostatic overload” [155]. Multiple biological markers for stress, like adrenaline, noradrenaline, adrenocorticotropic hormone (ACTH), dehydroepiandrosterone and vasopressin, can impact GnRH, LH, FSH, prolactin, cortisol, endogenous opioids and melatonin levels exerting potential, deleterious effects on fertility (Figure 3) [156]. In particular, the follicular levels of glucocorticoid hormones, such as lower follicular cortisone and a higher cortisol/cortisone ratio, have shown to exert a significant effect on pregnancy rates in IVF (Figure 3). A study including 291 women undergoing IVF/ICSI showed that a state of anxiety had a slightly stronger correlation (*p* = 0.01 versus *p* = 0.03) with treatment outcome than depression (Figure 3) [157].

There is ample evidence that personality characteristics, coping modes, stress susceptibility and resilience correlate with IVF outcomes and that acute and chronic stress affects fertility [158,159,160]. Interestingly, the treatments for infertility can by itself cause additional stress, and acute stress after discovering infertility issues despite being different from chronic stress may also affect IVF outcomes (Figure 3) [161]. Thus, psychology may help to reduce infertility due to acute and chronic stress and should be the first option before more invasive steps are taken.

#### 4.2.3. Sleep

The impact of sleep on fertility is still not completely understood, the relationship between sleep and fertility is clearly a complex one [162,163]. On one side, sleep disturbances are able to induce alterations of the hormones that play a major role in human reproduction [164]; on the other, infertility treatments, increasing infertile couples’ stress, lead to sleep disturbances which, in turn, affect the course of the treatment. It has to be clarified that “sleep” is a blanket term and the different aspects of sleep, such as bed-time, duration of sleep, quality of sleep, and the influence of circadian rhythm, can all adversely impact both male and female fertility.

With regard to male infertility, semen quality in terms of sperm count, motility, and viability, and male reproductive hormones have been evaluated in relation to sleep. Late bedtime, short sleep duration and poor quality of sleep negatively impact semen quality (Figure 3) [165]. Jensen et al. [166] observed an inverse u-shaped relationship between sleep quality and semen quality [166]. Their findings were supported by the observations of Green et al. [167] who added that bedtime usage of smartphones and tablets was negatively associated with semen quality [167].

The possible mechanisms that lead to these impairments are: (i) the disruption of circadian rhythm that dysregulates the circadian locomotor output vycles kaput genes [165]; (ii) elevated levels of anti-sperm antibodies (ASA), in individuals with short sleep duration [165]; (iii) the decreased levels of testosterone that disrupts the spermatic cycle maintenance [168]; and (iv) the increased expression of apoptosis-related nitric oxide genes in the testis associated to sleep deprivation reduced sperm viability and motility [169].

Sleep affects also various aspects of female fertility and pregnancy. Women who had a mean sleep duration less than five hours experienced an increased risk of menstrual cycle irregularities, compared to those who had more than eight hours of sleep every day [170]. Another study reported that women who had less than six hours of sleep every day and experienced trouble sleeping had a relatively lesser fecundability rate compared to women who slept 8 h per night and had no trouble sleeping (Figure 3) [164].

Irregular menstrual cycles and increased risk of miscarriages were observed in women who worked night shifts, suggesting that, apart from the quality and quantity of sleep, disruption of the sleep cycle also impacts fertility [164]. Shift work causes circadian dysrhythmia which interferes with fertility by altering the secretion of reproductive hormones, increasing insulin resistance and inflammation. Studies on murine models have reported that circadian rhythm disorders have an impact on fertility, independent of the hypothalamic-pituitary axis [170]. Several mechanisms may be involved in this process. First, the activation of the hypothalamic-pituitary axis (HPA), observed in chronic insomnia, may independently affect the reproductive capacity [171]. Another important factor is the disruption of the hormonal milieu necessary for reproduction. Indeed, acute sleep deprivation increases the TSH secretion, whereas extended sleep deprivation diminishes the TSH levels. Partial or total sleep deprivation increases the LH and oestradiol levels, and profoundly suppresses the prolactin release. All these variations affect the key steps leading to proper fertility, such as successful ovulation, conception, and implantation [168]. Sleep loss and poor sleep quality are associated also with an increase in inflammatory markers, such as TNF, IL-6, and CRP. This supports the hypothesis that excess oxidative stress and compromised immunity due to sleep problems can affect fertility [168,171]. Short sleep is also significantly associated with chronic diseases, such as obesity, type 2 diabetes, and cardiovascular diseases which can also influence fertility [170]. 

Interestingly, sleep alterations have been related also to assisted reproduction techniques outcomes. About 35% of women seeking fertility treatment and undergoing intrauterine insemination (IUI) had sleep disturbance, and 43% of women receiving IVF experienced ‘troublesome sleep’ [164,172]. The psychological stress associated with the treatment can cause sleep deprivation, which in turn exacerbates sympathetic activation, oxidative stress, and changes in reproductive hormones. These factors are detrimental to treatment outcomes and can reduce the probability of a successful procedure. Goldstein et al. [173] observed a linear association between baseline total sleep time and oocytes retrieved, which is an important outcome in IVF. The number of oocytes retrieved increased with an increase in total sleep time suggesting that sleep duration can affect the success of IVF [173].

Lifestyle factors that affect fertility have the advantage that once diagnosed, they are modifiable, thereby increasing the chances of conception. Similarly, sleep-related factors such as early bed-time and sleep duration are patient-centric factors that can be addressed without the need for any pharmacological treatment. In fact, sleep disorders such as obstructive sleep apnea, when treated with continuous positive airway pressure therapy, resulted in a decrease in prolactin levels and improvement in fertility [174], demonstrating the reversibility of the negative effects of sleep disorders on fertility.

### 4.3. Food and Nutraceuticals

Recently, a lot of attention has been devoted to the role of nutrition as a possible causative factor of both female and male infertility. Numerous studies have been conducted to identify the dietary factors that can affect fecundity and to pinpoint the most effective nutrients to be considered by couples seeking pregnancy [175]. As a general note, food does not have a direct or immediate effect on fertility. The issue should be rather approached by understanding how several individual factors add up and conjure to have a positive or negative return. In other words, there is no single nutrient that can harm or promote fertility: it is what we eat day after day, meal after meal, that makes the difference. This means that a generally poor diet cannot be counterbalanced by the uptake of beneficial nutrients. Similarly, a small deviation from a proper and healthy diet will not deeply affect your fertility levels.

Nevertheless, nutrition is so important for fertility that hundreds of scientific studies exist on the subject [175]. This has prompted experts throughout the world to create guidelines, applicable to both men and women, that have shown how dietary modifications can improve the odds of conception. The obvious implication of nutrition on female fertility can be easily assessed by the correlation between an adequate amount and a correct distribution of body fat with an higher probability of conception. Contrarily, women that are underweight due to a lack of nutritional intake, and overweight or obese women in which insulin resistance and increased androgens production due to fat have fewer ovulations over the course of a year. Just like for women, also in males obesity can reduce fertility due to the increase in estrogens derived from the conversion of androgens in the adipose tissue. Further, the free radicals that are present as a consequence to excessive energy production can elicit oxidative damage of the sperm. For similar reasons, several metabolic diseases such as diabetes, hypertension, and dyslipidemia can induce damage due to excessive fat [175].

**Figure 4:**
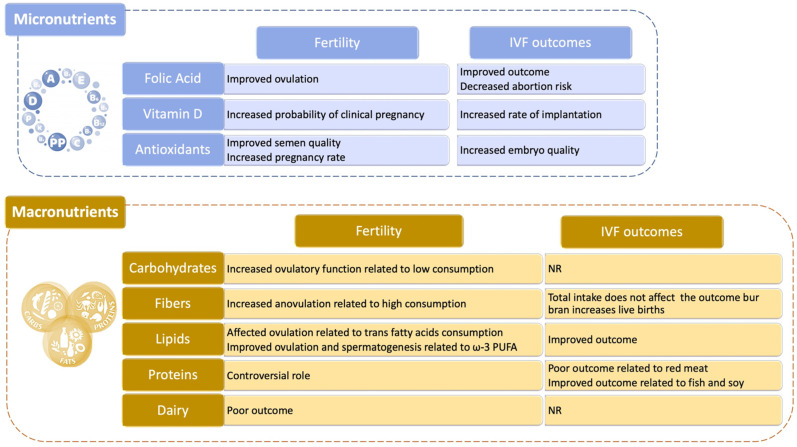
Role of nutrition and dietary habits on human reproduction. Both micro- and macronutients are able to influence different aspects of male and/or female infertility and to influence also the outcome of in vitro fertilization (IVF) procedures [175,176,177,178,179,180,181,182,184,185,186,187,189,190,191,192,194,195,198,199,200,202,203,205,206,207,212,214,215,216,217,218,219].

While our effort cannot possibly encompass all the studies available, in the following paragraphs we made an attempt to analyze the role that various food categories have on female and male fertility, dividing them in two classes: micro- and macronutrients (Figure 4).

#### 4.3.1. Micronutrients

Folic Acid. According to several studies, folate can improve the chances of success for couples undergoing IVF as it helps to increase the number of oocytes available for fertilization techniques, embryonic quality and pregnancy rate [176,177,178]. There is also extensive data relating to natural pregnancies according to which folic acid supplementation leads to a reduction in the risk of spontaneous abortion [179]. Further confirming these conclusions, folate supplements users had approximately one-third lower risk of developing ovulatory infertility compared to those who did not take multivitamins [180]. Similarly, folate intake was related to a lower frequency of episodic anovulation in a prospective study of young healthy women [181] and to a shorter time to pregnancy in a large Danish cohort [182]. In another small randomized controlled trial of subfertile women, women who took a daily multivitamin (containing 0.4 mg folic acid) had a 16% higher probability of pregnancy than the placebo group [183], and a study focusing on assisted reproductive technology’s (ART) outcomes revealed that women consuming >0.8 mg/d folate compared with those consuming <0.4 mg/d, before conception, had a higher probability of live birth [178].

Vitamin D. Vitamin D stimulates ovarian steroidogenesis, promotes follicular maturation, and regulates the mediators of successful implantation [184,185]; however, studies evaluating the relation between vitamin D and fecundity in healthy human populations failed to show a strong associations compared to nonhuman studies. In a meta-analysis of 11 studies, Chu et al. [186] found that women with adequate vitamin D levels, compared with women with either deficient or insufficient vitamin D levels, had a higher probability of clinical pregnancy and live birth [186].

Antioxidants. Among this wide class of molecules and compounds, coenzyme Q10, which is part of the electron transport chain responsible for generating energy in every cell of our body, may be regarded as one of the most promising for its positive role in rescuing oxidative stress-induced damages [187]. With regard to IVF outcomes, antioxidant supplementation may have beneficial effects in terms of quality and cryotolerance of in vitro produced embryos, together with positive effects on in vitro maturation oocytes and on early embryonic development. However, it is unknown what specific antioxidants or what doses are responsible for this benefit, suggesting that more studies are necessary to fully comprehend their effect on female fertility. Antioxidant supplementation appears to be helpful also for males and sperm health [175]. Supplementation with antioxidants and nutrients involved in the one-carbon metabolism pathway (folate, vitamin B12 and zinc) also appears to be beneficial [188]. A Cochrane review of randomized trials of antioxidant supplementation for men in couples undergoing infertility treatment found that antioxidant improved semen quality and clinical pregnancy rates [189].

#### 4.3.2. Macronutrients

Carbohydrates. An elevated consumption of food such as bread, potatoes, rice and pasta, which are rich in sugars, can induce an elevation of glycemia which in turns activate the pancreas to produce more insulin. This hormone, besides helping the body reduce blood sugar levels can also stimulate the production of testosterone that should be physiologically present only at very low levels in women. The more testosterone is generated the less likely is for a woman to conceive. A reduction in dietary carbohydrates among PCOS women can improve insulin sensitivity [190,191,192], and decrease circulating testosterone levels [191], potentially enhancing ovulatory function. In turn, it is recommended to increase the consumption of whole wheat, as whole grains and their constituents, such as phytic acid, vitamins, and selenium, have antioxidant, anti-inflammatory properties and positive effects on glucose metabolism, which may potentially boost fertility because insulin resistance and oxidative damage have been implicated in the pathogenesis of subfertility [193].

Fibers. A diet rich in fiber has been associated with an increased risk of anovulation in one study [194] but was unrelated to ovulatory infertility in the long run in another one. With regard to ART, total fiber intake was unrelated to its success, but the intake of bran was responsible for the positive association between whole grains and live birth rates described above [195]. 

Lipids. Fatty acids are used as energy substrates during oocyte maturation and early embryo development [196], and they serve as critical precursors for a variety of substrates that play a vital role in implantation and sustenance of pregnancy [197]. However, trans fatty acids increase insulin resistance [198], which adversely affects ovulation [199]. A study showed that consuming trans fat instead of carbohydrates or other unsaturated fat was associated with higher risks of ovulatory infertility [200]. Furthermore, trans fat intake was associated with reduced fecundability in a large North American study [201].

Contrarily, the intake of long-chain omega-3 polyunsaturated fatty acids (ω-3 PUFA) was associated with an increase in luteal progesterone concentration total estradiol levels and lower risk of anovulation [202]. There were favorable associations between ω-3 PUFA and reproductive endpoints also in ART settings, as shown in a small study of overweight and obese women undergoing ART, in which the preconception intake of ω-6 PUFA (especially LA) was associated with improved pregnancy rates [203]. Finally, the EARTH study reported that for every 1% increase in the blood levels of long-chain ω-3 PUFA, the probability of clinical pregnancy and live birth increased by 8% [204]. Taken together, higher ω-3 PUFA and lower trans fatty acid intake may enhance female fertility as well as spermatogenesis.

Proteins. Amino acids contained in meat, fish, eggs, milk but above all in the vegetable proteins of legumes are essential for fertility and should be taken at least twice a week also because their content of zinc and selenium have an anti-aging action that counteract the damage caused by oxygen free radicals and improve the quality of oocytes. Despite these positive effects, several concerns have arisen because of the potential for contamination of dietary protein sources by pesticides and endocrine-disrupting chemicals [205,206,207], and the presence of measurable amounts of steroid hormones and growth factors [208,209,210,211], that when absorbed, may alter reproductive outcomes. While, blastocyst formation following ART was decreased among patients consuming more red meat, blastocyst formation was positively affected by fish consumption [212]. Furthermore, there were strong relationships between fish intake and shorter time to pregnancy [213], as well as between ω-3 PUFA and higher fecundability among non–fish oil supplement takers [201]. This supports the benefits of consuming fish with low mercury levels and high concentrations of ω-3 PUFA [201,214]. On a similar note, in women seeking fertility treatments, soy isoflavone supplements were associated with improvement in reproductive outcomes such as increased live births [215], higher endometrial thickness and pregnancy rates after intrauterine insemination [216], and in vitro fertilization [217]. Similarly, dietary soy intake was positively related to the probability of live birth after ART among EARTH study participants [218].

Dairy. In human populations with higher per-capita consumption of milk, there was a steeper decline in fertility with aging [219]. However, consumption of three or more glasses of milk per day was protective of female fertility in an agricultural population in Wisconsin [220]. Nevertheless, dairy food intake was not related to any of the evaluated intermediate outcomes such as the response to gonadotropins, embryonic development, implantation, and clinical pregnancy.

## 5. Role of Epigenetics in Human Reproduction

As reviewed in the previous sections, several intrinsic and extrinsic factors contribute to human reproduction and may be involved in the onset of a disturbed reproductive status leading to infertility issues. Like for other complex traits, what is emerged is that the final reproductive phenotype of an individual is not dependent on a single factor but is rather the result of a complex interplay between genetic factors and the environment. In this view, epigenetic modifications, including micro RNAs (miRNAs) and DNA methylation, being influenced by the environment and lifestyle habits on one side and being able to modify genes expression on the other, may be a possible mechanism linking these two sides of the same coin. Accordingly, several evidences are accumulating regarding miRNAs- or methylation-mediated functions impaired in infertile individuals. 

In particular, miRNAs are a class of small non-coding RNAs able to regulate genes expression and thus involved in an increasing number of pathophysiological conditions [221]. Preliminary studies on mouse models have highlighted their contribution also to female infertility [222]. Next, miRNAs have been involved in the regulation of different aspects of reproduction, including germ cells proliferation, oocyte maturation, embryo and fetus development, also in humans [223]. As a consequence, miRNAs alterations have been reported in association to several reproductive conditions causing infertility, including polycystic ovary syndrome, endometritis, intrauterine growth restriction, and aging [224]. Furthermore, miRNAs have also been associated to sperm cell fertility since they have been involved in spermatogenesis and sperm maturation [225]. Even if the exact mechanisms responsible for these miRNAs alterations are not still completely understood, the possibility to evaluate specific miRNAs values in biological samples taken from patients is an attractive field for the development of novel diagnostic tests able to discriminate different reproductive disorders affecting both males or females.

DNA methylation is another epigenetic mechanism involved in gene expression regulation. Oocytes DNA methylation is a gradual process acquired during their maturation and follicle development [226]. This process seems to be required for oocyte development and competence: indeed, methylation allows for successful fertilisation and embryonic development [227]. In adddition, testicular germ cells have a highly unique pattern of DNA methylation [228]. Altered DNA methylation has been related to altered semen parameters and male infertility. Houshdaran et al. [229] showed that poor sperm concentration, motility, and morphology were associated with DNA hypermethylation in multiple loci [229]. Recently, a systematic review has focused on the actual knowledge regarding the role of DNA methylation in human spermatozoa [230]. Totally, 135 different studies were reviewed highlighting that male subfertility and alteration of some semen parameters, i.e., oligozoospermia, seem to correlate with altered spermatozoal DNA methylation, even if no reproducible data are reported by the different published papers [230]. This finding underlines how the sperm cell methylation status depends on several factors, including cigarette smoking, advanced age, and environmental pollutants. Thus, even if inconclusive data are reported so far, these studies strengths the link between intrinsic and extrinsic factors in reproduction and open the way to future, more robust studies aiming to address this issue definitively. 

## 6. Discussion

**Figure 5:**
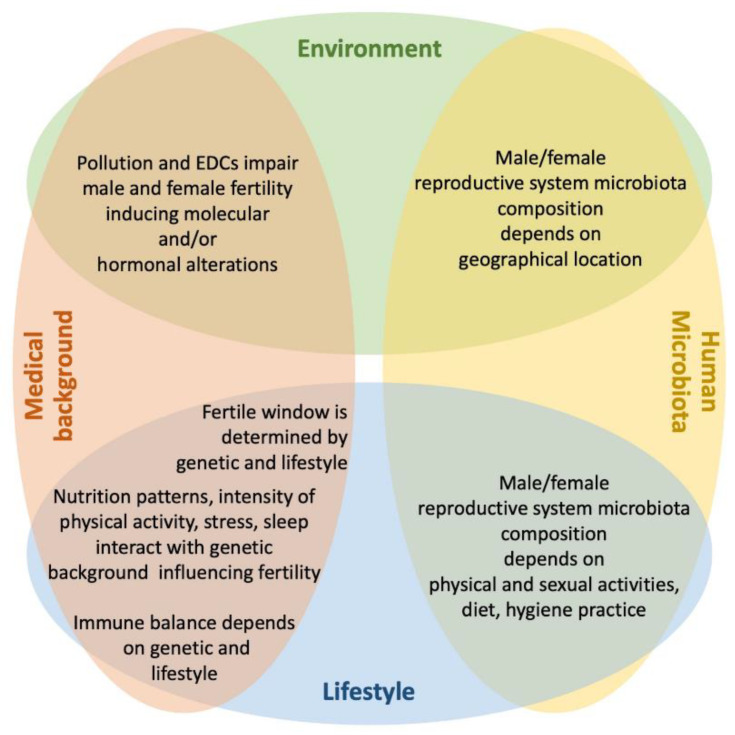
Intrinsic (microbiota and medical background) and extrinsic (lifestyle and environment) factors can have an impact on the overall state of reproductive health. It is interesting to note how what we can choose (i.e., physical activity, diet, habits) intrinsically affect our microbiota and can also modify what we have inherited (i.e., medical or genetic background). Moreover, even what we are exposed to (i.e., pollution and EDCs) modifies the microbiota composition and impacts the medical and genetic background (i.e., fertile window).

The “reproductive behavior” is something that depends on both biology and personal experiences (Figure 5). Studying genetics alongside social behaviors is becoming an increasingly popular approach. The sociogenome project was started exactly with this in mind: studying gene-environment interaction, or how genetics interacts with the environment and social factors [www.sociogenome.org (accessed on 16 March 2021)].

Through this new alliance, sociologists, demographers and geneticists have been able to include a larger number of variables, extending their studies to meta-analyses with increasing sample sizes and more significant outcomes. Many of the previous studies concerned female fertility were based exclusively on the availability of eggs, while for men most of the data came from the analysis of sperm samples. For such a limited set of variables, it was difficult to collect a lot of information. Taking a step back and using data from clinical studies linked to DNA, in which the demographic characteristics for all participants were also collected, made it possible to widen the spectrum of possible causative effects, ushering a new era of correlation studies between the environment and the genetic background.

Several meta-analysis studies performed on datasets including men and women have searched for the genes associated with the age of the first child and the total number of children, working on data concerning both genetics and behavior [55,56]. These studies discovered several genetic variants that play a role in explaining the age of the first child and the number of children, some of which are involved in mechanisms related to reproductive behavior. For example, the age of the woman at the first menarche and that of the menopause, but also variants associated with genes that play a role in endometriosis or polycystic ovary syndrome [231]. Thanks to the advent of sophisticated molecular techniques, especially in the last decade, this research area has made great strides forward.

The first studies on heredity were mainly based on the study of twins in order to understand, through specific statistical techniques, which part of the variability of a trait (or a pathology) could be explained by genetics. In recent years, genetic analyses have become more accessible and it has become possible to collect data on different genetic variants, thus allowing to understand the genetic and biological role of such variations. This type of research, known as genome-wide association study, investigates a large portion of the genes of different individuals, in the attempt to establish their genetic variants, and then associate these diversities with the traits of interest. In this case, the age of the first child and the number of children [56].

An interesting aspect of this research is that most of the genetic components identified are shared between men and women. The same genes that influence at what age a woman will have her first child (and how many children she will have) are the same that influence these aspects in men. It appears that in both men and women fertility is closely linked with age: some may have children up to an advanced age, while others begin to become less fertile much earlier. We still know little about what factors influence this difference, but studying it at the genetic level could permit to identify this subjective “fertile window”. This knowledge will also allow us to take steps forward in our understanding of infertility and in predicting the optimal timeframe to procreate. After all, in most Western countries, the birth rate is falling and couples are having children later in life. In such context, the genetic, behavioral and environmental components in explaining fertility are increasingly important to understand if a couple is at risk of early infertility [232].

Similarly, the relationship between nutrition and fertility can be addressed from two point of views. On one side, the way we eat day after day, from childhood to adulthood, affects our fertility in a positive or negative way. If we realize that our way of eating is not properly supporting sex hormones, ovarian quality and menstrual regularity, we can change course at any time. A change in nutrition patterns will not result in immediate effects, but within a few months it is possible to to see some changes. On the other hand, the most targeted diet therapies are the one aiming to correct endocrine and metabolic imbalances, the effect of which is also applied to fertility (for example, polycystic ovary syndrome, uterine fibroids, endometriosis). Several elements can have an impact on the overall state of reproductive health and these can be addressed by a change of dietary or lifestyle habits or by the inclusion of supplements. This has spurred the advent of a plethora of physical training routines combined with fertility diets that can lower the risk of ovulatory infertility [233] and increases the rates of sustained pregnancies [234]. 

As detailed in the previous sections, it is now clear that reproduction is a complex trait involving both intrinsic and extrinsic factors cooperating to ensure a proper fertility status. Identifying these factors and, more importantly, understanding how they are able to influence each the others within the same individual and between the two partners, not only will allow us to reach a better comprehension of human reproduction, but may also open the way to the development of more tailored and personalized interventions for fertility issues management. In this view, the integration of the “omics” with artificial intelligence machine learning systems, able to provide decisional algorithms by integrating different sources of data, will reinforce the transition to precision medicine. 

## 7. Conclusions

Despite the obvious anatomical differences and regardless of the female and male reproductive systems’ specific functions, one aspect that emerges as common between the two is the amount of information and effort that is devoted to their correct functioning. It is estimated that 10% of our genetic information is used to guarantee the proper working of human reproduction. When so much information is involved, some of it can go missed in translation, increasing the probability of communicating the wrong instructions. When it comes to the reproductive system, the scenario gets even more complicated by the interplay between the underlying genetics, the external factors, and our commensal microbiome which can either exacerbates the outcome or mitigate the adverse effects. The growing amount of evidence supporting the interaction between genes, the environment and microbes, allowing a better understanding of these connections. This, in turn, will provide new avenues to approach the treatment of infertility.

## Data Availability

Not Applicable.
